# Bereavement support for family caregivers: The gap between guidelines and practice in palliative care

**DOI:** 10.1371/journal.pone.0184750

**Published:** 2017-10-04

**Authors:** Samar M. Aoun, Bruce Rumbold, Denise Howting, Amanda Bolleter, Lauren J. Breen

**Affiliations:** 1 School of Nursing, Midwifery and Paramedicine, Faculty of Health Sciences, Curtin University, Perth, Western Australia, Australia; 2 Palliative Care Unit, Department of Public Health, La Trobe University, Melbourne, Victoria, Australia; 3 WA Cancer and Palliative Care Network, Health department of WA, Perth, Western Australia; 4 School of Psychology and Speech Pathology, Faculty of Health Sciences, Curtin University, Perth, Western Australia, Australia; TNO, NETHERLANDS

## Abstract

**Background:**

Standards for bereavement care propose that support should be matched to risk and need. However, studies in many countries demonstrate that palliative care services continue to adopt a generic approach in offering support to bereaved families.

**Objective:**

To identify patterns of bereavement support in palliative care services based upon the experience of bereaved people from a population based survey and in relation to clinical practice guidelines.

**Design:**

An anonymous postal survey collected information from clients of six funeral providers in four Australian states (2014–15), 6 to 24 months after the death of their family member or friend, with 1,139 responding. Responses from 506 bereaved relatives of people who had terminal illnesses were analysed. Of these, 298 had used palliative care services and 208 had not.

**Results:**

More people with cancer (64%) had received palliative care in comparison to other illnesses such as heart disease, dementia and organ failure (4–10%). The support for family caregivers before and after their relative’s death was not considered optimal. Only 39.4% of the bereaved reported being specifically asked about their emotional/ psychological distress pre-bereavement, and just half of the bereaved perceived they had enough support from palliative care services. Half of the bereaved had a follow up contact from the service at 3–6 weeks, and a quarter had a follow-up at 6 months. Their qualitative feedback underlined the limited helpfulness of the blanket approach to bereavement support, which was often described as “not personal” or “generic”, or “just standard practice”.

**Conclusions:**

Timeliness and consistency of relationship is crucial to building rapport and trust in the service’s ability to help at post-bereavement as well as a focus on the specific rather than the generic needs of the bereaved. In light of these limitations, palliative care services might do better investing their efforts principally in assessing and supporting family caregivers during the pre-bereavement period and developing community capacity and referral pathways for bereavement care. Our findings suggest that bereavement support in Australian palliative care services has only a tenuous relationship with guidelines and assessment tools, a conclusion also drawn in studies from other countries, emphasizing the international implications of our study.

## Introduction

In many countries, the most coherent approach to bereavement support is provided by palliative care services, which emphasise the care of patients with terminal illnesses and their family carers before and after the patient’s death. Policies and guidelines on standards of care propose that supports should be offered according to need [1–4]. However, despite such policies and guidelines, studies demonstrate that palliative care services in general adopt a blanket approach to supporting bereaved families regardless of risk or need [5–7]. For instance, one survey of Australian palliative care services determined that 95% (of 236 services) offered some form of bereavement support [8], with the most common types of support being a telephone call (offered by 86%), memorial service, (66%), letter (55%), anniversary card (53%), group sessions (31%), information package (5%), and informal gatherings (4%). Similar eclectic approaches appear to be taken in other countries such as the United States, Canada, the United Kingdom, and Japan [9]. A recent survey of bereavement support practice in 25 European countries (370 palliative care services) [10], showed that bereavement care was not an integral part of palliative care in a substantial number of palliative care services; more than two thirds of services did not use formal guidelines or standards to inform their bereavement care; while only about half the services employed a full time, part time or hourly bereavement coordinator or provided formal training in bereavement care.

Furthermore, where risk assessments were made, the usefulness of these assessments varied widely and often depended upon the subjective opinion of service providers or the use of non-validated screening tools [8, 11]. Approximately two-thirds of the services reported engaging in some form of bereavement risk assessment, with two-thirds relying on multidisciplinary team opinion, more than half using a formal tool to assess bereavement risk, and approximately half relying on a single staff member’s opinion (some services reported using more than one method) [8].

The situation is complex as there is no clear evidence to guide the development and allocation of cost-effective bereavement support services [8, 9, 12]. A core dilemma in making bereavement risk assessments is that much of our current evidence comes from groups of bereaved people who have self-referred, either to a community counselling service or in response to an invitation from a palliative care service [13]. The resultant focus on professional support obscures alternative strategies that could be employed. Accordingly, we know less about bereavement experiences and needs of people who did not use palliative care services following an expected death, and considerably less again about the bereavement experiences of those for whom the death was unexpected.

We can, however, see that offering professional bereavement support, irrespective of need, to all people bereaved through deaths in palliative care is neither effective nor affordable [8, 9, 12, 14]. In Australia, 159,052 deaths were registered in 2015 [15], with the cause of death for at least two-thirds being a chronic degenerative illness. About 35,000 were in receipt of palliative care [16], but many more could have been eligible. For example, a large, retrospective cohort study in one Australian state reported that only about 60% of the population, whose dying should have been amenable to palliative care, actually received palliative care services [17].

Recognising these challenges, we embarked upon a population based survey of bereavement experiences and support needs to inform practice and develop a model of care that might apply to a whole community, not only to clients of a palliative care service. The three-tiered public health model of bereavement support we developed aligns intervention with need and is compatible with bereavement care standards and policies [12]. This model identifies three risk groups: a low risk group of bereaved people that are likely to adjust in time with appropriate support from family and friends; a moderate risk group that would benefit from grief counselling or a volunteer-led, or peer support group, to prevent the development of ongoing complications; and a high risk group that would most likely require formal support from health professionals. Empirical support for this model has been demonstrated [18]. This model challenges current palliative care bereavement provision in two ways. It suggests that the bulk of bereavement support should be located in local communities, via people’s existing social networks [19], and it questions strategies for offering bereavement care that pre-dispose bereaved people to look for support in professionalised health services, such as palliative care, more than their local community.

### Aim

The aim of this study is to identify patterns of bereavement support in palliative care services based upon the experience of bereaved people from a population based survey and in relation to clinical practice guidelines.

### Objectives

The objectives are:

- To compare the profile of those who were cared for by a palliative care service with those who did not
- To compare the profile of the bereaved whose family member used palliative care services with those who did not
- To compare the bereavement support reported by the bereaved in relation to the clinical practice guidelines
- To describe which bereavement support practices were found helpful, or not helpful, by the bereaved
- To compare sources of bereavement support reported by those who did and did not receive palliative care

## Methods

Ethics approval was granted by Curtin University Human Research Ethics Committee (HR-57/2012).

An anonymous postal survey was used to collect information from clients of six funeral providers in four Australian states (2014–15), 6 to 24 months after the death of their family member or friend. A total of 6,258 study packages were delivered to the six funeral providers who agreed to participate in the study. These packages contained an invitation letter addressed from the funeral provider to the family, information sheet, the questionnaire, a list of support services for the family to use in case the participant became distressed while completing the questionnaire, and a reply-paid envelope. The funeral providers selected from their databases clients who were bereaved 6–24 months ago, attached names and address labels on the envelopes and mailed the study packages. Consent was implied by the return of the completed survey. No reminder letter was sent as the funeral providers felt it was too intrusive for the bereaved families. Clients were eligible to participate in the study if they had been bereaved by the death of a close family member or friend in the specified timeframe, were able to read, understand and write in English, and were over 18 years of age.

The questionnaire comprised 82 questions divided into 8 sections [20]. This present article focuses mainly on the section addressing the bereavement support received from a palliative care provider. At the start of this section, a lay definition of palliative care was provided so respondents were clear about whether they needed to complete this section: “*Palliative care is provided to patients with life-limiting/ terminal illness to ease symptoms and improve quality of life and support their families”*. Some of the questions in this section were aligned with the clinical practice guidelines for the psychosocial and bereavement support of family caregivers of palliative care patients [21]. Although these 20 guidelines were framed from a service provider perspective, we asked the questions from the bereaved people’s perspective regarding five of these guidelines (numbers 8, 12, 13, 17, 19). Questions on perceived support were adapted from the VOICES survey [22] and one question on level of caring was adapted from the Omnibus survey [23].

### Analysis

Descriptive statistics for variables were calculated: frequencies and proportions for categorical variables; means, standard deviations, medians, minimums and maximums for continuous/discrete variables. Significance testing was performed using chi-square for categorical variables, and nonparametric tests for the median for the non-Normally distributed continuous variables. Significance was set at the p = 0.05 level, and analyses were conducted using IBM SPSS Statistics Version 24. The open ended responses were manually coded using an open content analysis process [24].

## Results

**Fig 1:**
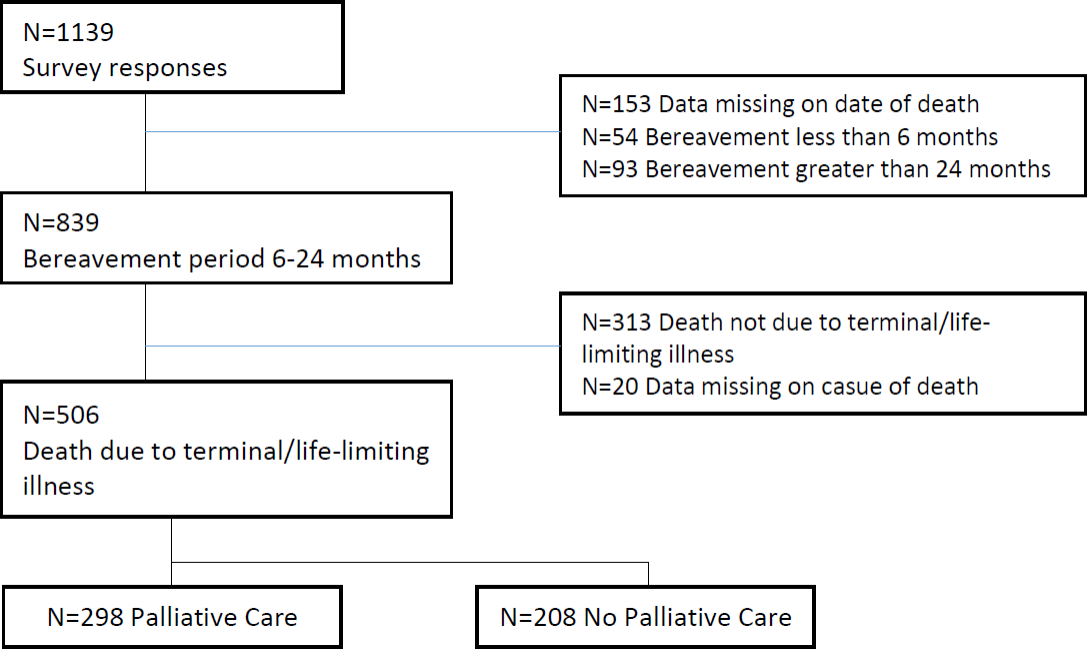
Flowchart of participation.

One thousand one hundred and thirty nine individuals completed the survey with a mean response rate of 18.1% (ranging from 13.3% to 28.6% between the six funeral providers). Those who had shorter or longer bereavement period than specified a priori; those who did not die from a terminal illness; and those who did not provide a date of death or cause of death were removed from the analysis. The sample was consequently reduced to 506 individuals. Of these, 298 had experience with palliative care services (PC) and 208 did not (NPC) (Fig 1).

### Profile of the deceased

Half of the total deceased were female with a mean age of 77 years (SD 13.9), and those in the NPC group were slightly older (79 vs 76 years). Other characteristics that differed significantly between those who received palliative care versus those who did not were the type of terminal illness: the PC group had predominantly cancer (64.1% vs 26.4%, p<0.001) and the NPC group had more heart disease, dementia, lung disease and organ failure (Table 1). The PC group were significantly more likely to have an advance care plan (17.4 vs 6.1%, p<0.001) or an advance health directive (13.9 vs 7.1%, p<0.006) than the NPC group.

**Table 1:** Profile of the deceased (%).

	Total	Palliative Care		
	n = 506	PC	NPC	p-value
Gender (Female)	49.0	47.5	51.4	NS
Mean Age in years (SD)	77.2 (13.85)	76.0 (14.37)	79.0 (12.89)	0.015
Advance care plan	12.7	17.4	6.1	<0.001
Advance health directive	11.9	13.9	7.1	0.006
Cancer	48.6	64.1	26.4	<0.001
Heart disease	13.8	10.4	18.8	0.008
Dementia	12.5	8.1	18.8	0.004
Lung disease	6.1	4.0	9.1	0.006
Organ failure	11.5	7.7	16.8	0.001

### Profile of the bereaved

The majority of the bereaved who responded were female (70%), had mean age of 64 years (SD 11.5), 42% were married and 44% were spouses of the person who had died. The two groups differed in some characteristics: the NPC group had more ‘other relatives’ who have provided care than members of the immediate family, the median length of caring was twice as long (48 vs 24 months), care was described as not hands on but still close, while more day to day hands on care was provided by the immediate family members in the PC group (Table 2).

**Table 2:** Profile of the bereaved (%).

	Total	Palliative Care		
	n = 506	PC	NPC	p-value
Gender (Female)	69.8	72.7	66.2	NS
Mean Age in years (SD)	63.9 (11.46)	63.2 (11.58)	64.8 (11.26)	NS
Marital status (Married)	45.3	42.4	50.0	NS
Relationship (Spouse)	40.9	44.3	36.1	NS
Relationship (Other relative)	9.7	6.7	13.9	<0.001
Day to day hands on care	50.0	56.6	43.2	<0.001
No hands on care but still close	13.4	8.8	21.1	<0.001
Median length of care (month)	36	24	48	<0.001

### Experience of support pre- and post-death

The deceased received palliative care during their illness for a mean 3.7 months or median 1.0 month (range 0.03–96). Just over half of the bereaved felt that the care received by their relative/friend from the palliative care service in the last 3 months of life was excellent (53.1%), 31.0% felt it was good, 9.5% fair, and 4.4% thought the service was poor.

Of the group who received support from palliative care providers after the death of their relative/friend, 51.2% felt they got as much help and support as they needed, a third did not feel they received enough support and 13.2% stated that they did not need support. When asked their opinion of the support they received from all sources (health and community services and networks), a higher proportion, 68.2%, felt they had received enough support, reinforcing the role of the community based networks. The two groups (PC and NPC) did not differ in their perceived support from all sources.

**Fig 2:**
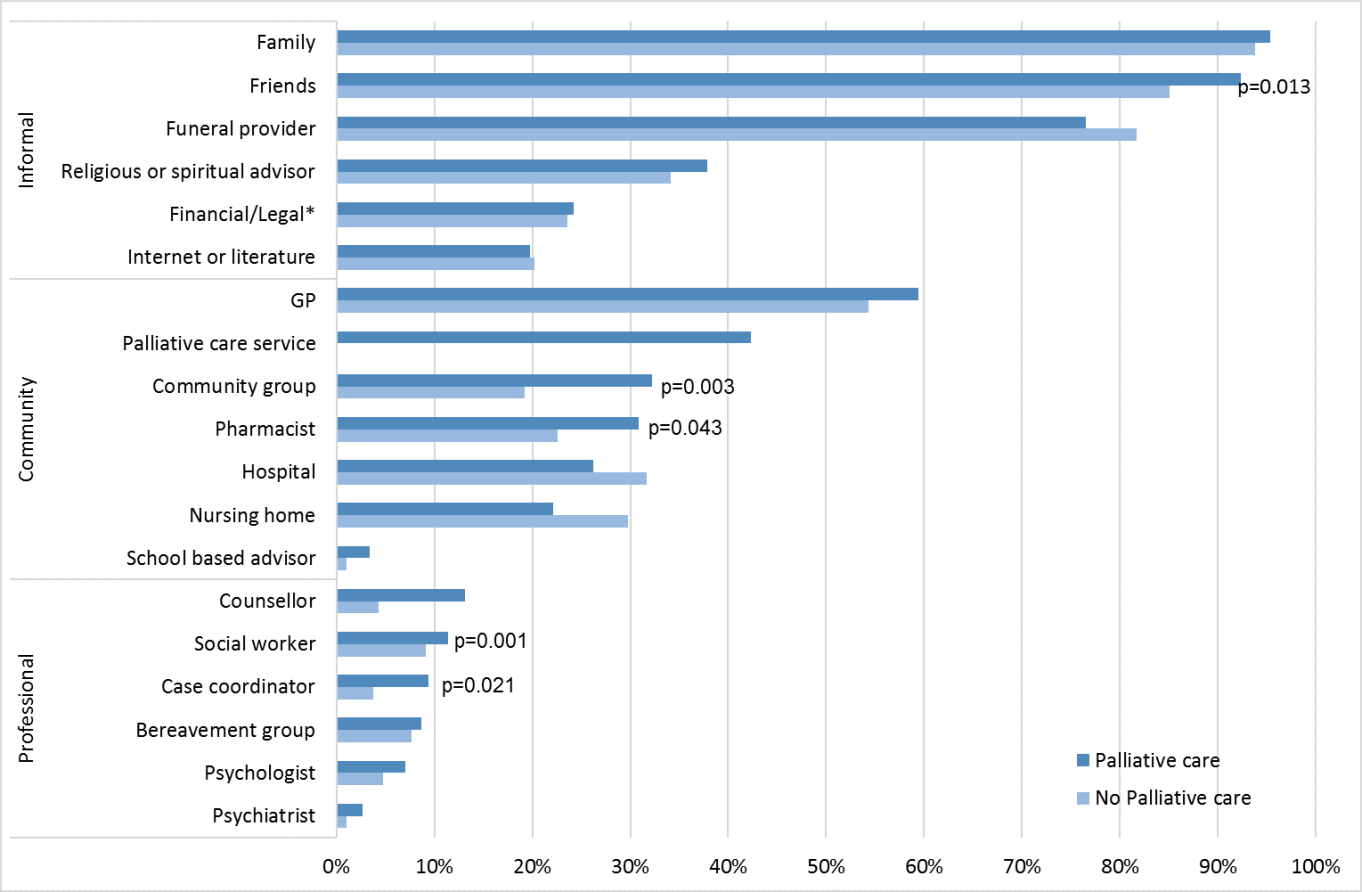
Sources of bereavement support accessed by respondents whose care recipients received or did not receive palliative care.

Fig 2 compares the sources of support for the two groups (PC and NPC). It seems that the PC group used more of all forms of support except the structured services of funeral provider, hospital and nursing home which were used more by the NPC. Professional sources, especially counsellors, were more frequently accessed by the PC group.

### Comparison to best practice guidelines

Table 3 outlines this support from the palliative care services as it relates more specifically to the best practice guidelines. The aligned survey questions were adapted to lay people. While three quarters of respondents were asked by staff how they were coping pre-death in general, only 39.4% reported receiving a needs assessment that covered the palliative care domains and more specifically were asked about the emotional and psychological domains. However 79.3% felt the staff kept them informed of the changes in the condition of the dying relative so they can be prepared for the death and 62.9% were offered information about grief and bereavement services. About half were contacted within 3–6 weeks of the death, 28.1% about 6 months after the death, and 25% at any other time.

**Table 3:** Experience of support from palliative care services in relation to the best practice guidelines.

Guidelines (Hudson et al, 2010)	Aligned questions from the survey	Percent agreeing N = 298
*Guideline 8*: *Conduct a needs assessment with the family caregiver(s)*. *This should include psychological and physical health*, *social*, *spiritual*, *cultural*, *financial and practical elements*.	Did staff ask how you were coping before the death of your relative/friend?	74.6%
	Did staff ask if you had experienced any significant stress, emotional or psychological problems, before the death of your relative/friend?	39.4%
*Guideline 12*: *When death appears imminent*, *ensure the family caregiver(s) are aware and assess preparedness for death*	Did staff tell you about changes in your relative/friend’s condition so that you were more prepared for his/her death?	79.3%
*Guideline 13*: *Confirm with the family caregiver(s) the type of support they may desire in the lead-up to death (e*.*g*. *last hours*, *days) and/or immediately after*	Were you offered information about grief and bereavement services that might be helpful following the death of your relative/friend?	62.9%
*Guideline 17*: *Contact the family caregiver(s) and other family members (as appropriate) to assess needs at three to six weeks post-death and adapt bereavement care plan accordingly*.	Did staff contact you (by phone, post or home visit) within 3–6 weeks of the death to find out how you were coping, and discuss any questions you might have had?	50.9%
*Guideline 19*: *Conduct a follow-up assessment of the family caregiver(s) and other family members (if appropriate) six months post-death*.	Did staff contact you (by phone, post or home visit) about 6 months after the death to find out how you were coping?	28.1%
	Did staff contact you (by phone, post or home visit) at any other time?	25.0%

### Differences in palliative care settings

Comparisons were undertaken between three settings: community palliative care, in-patient (79.5% hospital and 20.5% hospice/palliative care unit), and nursing homes. In terms of the quality of care received, there were significant differences (p = 0.001) between the three settings: More respondents rated in-patient settings as excellent/good (93%) followed by community (81%), with the least being nursing homes (73%) (Table 4). There were no significant differences in the perceived bereavement support between the three settings, although there was more unmet need in nursing homes (Table 5). Community palliative care did the most bereavement follow-up at 3–6 weeks (63%) followed by in-patient (58%) with nursing homes the least (31%), and differences were significant, p<0.001(Table 6). At the 6-months follow-up contact, the difference between community and inpatient was not pronounced (32–33%), while only 17% reported having a follow-up from nursing homes, and differences were significant, p = 0.003 (Table 7).

**Table 4:** Quality of palliative care received (n = 263).

	Excellent/Good		Fair/Poor		Don't know	
	n	%	n	%	n	%
Community Palliative Care	48	81.4	10	16.9	1	1.7
In-patient	124	93.2	8	6.0	1	0.8
Nursing home	49	73.1	15	22.4	3	4.5

F–Fisher’s Exact Test (chi-square). P-value = ***0*.*001***^***F***^

**Table 5:** Bereavement support from palliative care services.

	Enough support		Not enough support		Did not need support		Other	
	n	%	n	%	n	%	n	%
Community Palliative Care	34	55.7	17	27.9	7	11.5	3	4.9
In-patient	78	58.6	34	25.6	16	12.0	5	3.8
Nursing home	27	40.9	25	37.9	10	15.2	4	6.1

F–Fisher’s Exact Test (chi-square). P-value = 0.391^F^

**Table 6:** Bereavement follow-up contact at 3–6 weeks.

	Yes		No		Unsure	
	n	%	n	%	n	%
Community Palliative Care	37	62.7	21	35.6	1	1.7
In-patient	76	57.6	50	37.9	6	4.5
Nursing home	21	31.3	45	67.2	1	1.5

F–Fisher’s Exact Test (chi-square). P-value***<0*.*001***^***F***^

**Table 7:** Bereavement follow-up contact at 6 months.

	Yes		No		Unsure	
	n	%	n	%	n	%
Community Palliative Care	20	33.3	40	66.7	0	0.0
In-patient	42	31.8	81	61.4	9	6.8
Nursing home	11	16.7	55	83.3	0	0.0

F–Fisher’s Exact Test (chi-square). P-value = ***0*.*003***^***F***^

### Feedback on the usefulness of the bereavement follow-ups

Respondents were provided with an opportunity to comment on their responses in terms of how this follow up support was helpful or unhelpful. One hundred and seventy seven responses were provided or 59.4% of those who received palliative care. Participants who drew comfort from the 3–6 week follow up commented that they felt secure knowing that these services were available and appreciative of being remembered by the palliative care providers. The information on bereavement services was deemed relevant by some, and the services were appreciated as per comments such as *“the phone calls I received saved me from falling to pieces”* and *“I could not have coped without it”*. Some were ambivalent about the *“friendly visit- neither helpful nor unhelpful”* or it was *“helpful*, *although it felt like it was their standard practice”*.

Some respondents felt negative about their experience with the 3–6 weeks follow up, apparently because they did not have any contact and felt forgotten *“Post death*, *non-existent”*, *“after my wife’s death I was all on my own*. *Out of sight out of mind”;* or because the contact did not lead to further help *“it was just a phone call with no help offered”*, *“was just a query*, *no help”*, *“would have liked more what to expect*”. A number reported that when they received a call, it was from someone they had not previously met: *“Not helpful*, *unspecific call from someone I had never met”*, *“helpful if having the same nurse*. *Different people at each time is adding up to the stress and suffering”*. One respondent summarised the general feeling post-bereavement that *“there was a sense that ties with the palliative care provider were abruptly cut”*.

The feedback regarding the 6-month follow up showed that some of the bereaved appreciated the contact, *“the call was helpful to talk through my feelings and thoughts” or “this was helpful to talk to someone away from the family”*, or the invitation to a memorial service at 6 months. However many did not receive any follow-up (72%), *“no contact so don’t know if it could have been helpful or not”* and those who did receive a contact, described the support again as generic “*only a short phone call*. *That was it”*. Some did not need the service because they were contented with the support they were receiving from their funeral provider (mentioned quite often) or from their informal networks or organisations such as the Leukaemia Foundation and the Cancer Council: “*No we didn't need this service because we received bereavement counselling from [funeral provider]”; “funeral home sent card at 12 months*. *We were touched that they did”; “The offer of support was generic not necessarily personal but I was receiving support via family*, *relatives*, *friends and neighbours”*. In fact one respondent thought one more phone call was too many: *“I really didn't need another call*. *I would have thought it to be overkill*. *One phone call was enough”*.

## Discussion

This is the first study to report on the bereavement support experience of a community based sample. Most studies specific to bereavement support in palliative care to date have relied on clinical and service based samples [5, 7, 8, 25] which exclude people who did not use palliative care. Moreover, this is the first study to recruit from funeral providers’ databases, an innovative way to engage potential providers of bereavement support and which allows comparison between those who used palliative care with those who did not.

The difference in the profiles of those who did or did not receive palliative care has important implications for service delivery as we often do not know about those who did not use the service. In this study, from the terminal illnesses that lend themselves to palliative care, more people with cancer (64%) had received palliative care in comparison to other illnesses such as heart disease, dementia and organ failure (4–10%). These non-malignant diseases are still under-represented in palliative care ten years on from the study by McNamara, Rosenwax, & Holman [26] where it was reported that less than 10% of people who died of non-malignant diseases had accessed specialist palliative care services, compared with 66% of people who died of cancer. Yet these conditions, as described in this study, have required a longer period of care, twice as long as that of malignant diseases (48 vs 24 months, p<0.001), with more “other” relatives helping the immediate family (mainly spouses and adult children) in this prolonged period of care. Having a network of such caregivers may be a consequence of a longer disease trajectory in non-malignant diseases that allows networks of close friends and extended family to form. Burns et al [27] have pointed to this network of extended family and friends at the end of life, a network invisible to the health system but whose members also need adequate support in their role.

Those who received palliative care were 2–3 times more likely to have an advance care plan or advance health directive, albeit still in quite low proportions overall (12–13%), though a rate close to the 14% reported by an Australian study [28]. Internationally, the rate reported in the literature varies from 10% in American hospitals [29], to 16% in the Dutch general older population [30] and up to 25% among Swiss palliative care patients [31]. These rates are low despite studies showing positive impacts on patient quality of dying and reduction in stress and anxiety of families [32]. Many factors related to the health care professional, the patient/family and the health system are responsible for such poor end of life communication [32]. While bereavement is not addressed directly in advance care plans, it seems reasonable to assume that the existence of an advance care plan indicates some communication within the person’s social network about end of life wishes. Clarity about a dying person’s awareness of the situation, and an indication of preparedness, seem to be important for their family and friends in experiencing bereavement. The relationship between advance care planning and bereavement outcomes is important to explore in future studies.

The significant differences between the settings obtained in this study shed some light on the apparent discrepancy between pre- and post-death contact. Bereavement follow up tended to be common in community programs, less common in hospital programs. The site of palliative care affects follow-up, as one factor is privacy legislation. Because community palliative care providers tend to define the ‘patient and family’ as the unit of care, the service tends to keep better records of dependants and also has the ‘right’ to contact them after the death. Hospitals frequently lack adequate contact details for dependants, and are more reticent in following up because of concerns (often misplaced) regarding privacy legislation. However, in terms of the quality of care received pre-death, our results are similar to Pidgeon et al [33] who reported that family carers were likely to be less satisfied if receiving care from a community palliative care service.

Perceived bereavement support from all sources for PC and NCP groups are effectively the same, although there are differences in the patterns of support for the two groups (Fig 2). Possible explanations for the PC group’s greater access to resources, including professional services, are, first, that many palliative care patients have followed a complex care pathway which mobilises a multi-disciplinary team both prior to and whilst in palliative care: that is, family and friends are introduced to a wider range of sources of support by the nature of the illness and the service structure. Secondly, palliative care is usually intentional about encouraging caregivers to look for support, whereas hospitals and nursing homes are less likely to do this. That is, palliative care recognizes the needs of caregivers and normalizes asking for support in a way that other services may not. This is supported by Bergman et al [34] who reported that bereaved caregivers who used hospice care were more likely to have access to information and services not routinely available to non-hospice users.

This is also the first study to provide an insight into the application or practice of the guidelines by palliative care services from the service users’ perspective. While the majority of the bereaved (84%) felt that the care received by their relative/friend from the palliative care service was excellent to good, the support for themselves before and after their relative’s death was not considered as optimal. Clearly the systematic assessment of family caregivers’ support needs in the lead up to the patient’s death is not given enough attention by palliative care services. Only 39.4% of the bereaved reported being specifically asked about their emotional/psychological distress pre-bereavement; rather it was a more general question about coping (75%). Furthermore, following their relative’s death, just half of the bereaved perceived they had enough support from palliative care services. Half of these people had a follow up contact from the service at 3–6 weeks, and a quarter had a follow-up at 6 months. This lack of attention to members of the dying person’s social network in the time prior to the death suggests that little attention is being paid to links between the pre- and post-death experience of family and friends. This link, sometimes conceptualised as preparatory or anticipatory grief, deserves further attention [35].

It is evident that supporting family caregivers, while caregiving, has benefits pre- and post-bereavement: Results of the Carer Support Needs Assessment (CSNAT) trials in Australia have showed a significant reduction in caregiver strain during the caregiving period in community palliative care [36]. In another Australian study, family caregivers of older people discharged home from hospital were significantly more prepared to provide care and reported reduced caregiver strain and distress compared to family caregivers in the control group [37]. A larger trial of CSNAT in the UK [38] found a small reduction in grief, improvements in mental and physical health post-bereavement and in the probability of death at home.

Our findings, supported by the evidence from the literature, reinforce the need for palliative care services to take action during the pre-bereavement period to effectively assess and support family caregivers. Yet the ‘window of opportunity’ for contact with caregivers to assess their grief and bereavement needs while heading to the care recipient’s impending death does not seem to be well utilised in the palliative care system, although this is the only time caregivers are likely to have face to face contact with staff [11]. Clearly, a short length of stay with a palliative care service (median of one month reported in this study) is not conducive to building rapport with the family to prepare them for the death. In the post-death period, contact with bereaved caregivers is even more difficult for services due to various barriers such as staffing, funding, and availability of service contact with them [11]. This was quite evident in this study where half of the bereaved were followed up to 6 weeks and this proportion dwindled to a quarter at 6 months. Similarly Ghesquiere et al [39] have found that hospice bereavement support only reached half of the bereaved in their study, suggesting a need to improve care access and delivery.

The qualitative feedback from bereaved people has reinforced first-hand the limited helpfulness of the blanket approach to bereavement support, where a phone call or invitation to a memorial service did not address all needs, as one size does not fit all [9]. Some of the respondents have described their service as “not personal” or “generic”, or “just standard practice”, and some clearly did not see benefits in the contact because it was not tailored to their own needs or it did not occur at the time they most needed it. More importantly, some considered it stressful to receive calls from staff members they did not know, and these were different at every contact. So it seems for some who did receive a follow up, it was generally not helpful or neutral. We don’t know if this was because assistance at this time and from this source were not helpful or that it would be helpful if the quality was better. The responses also seem to point to differing expectations of different providers. A palliative care service follow-up call can be seen as standard practice, while the equally-standard practice of an anniversary card from a funeral provider can be embraced as an expression of care. It may also be that the person-centred services of community palliative care programs elicit higher expectations of bereavement follow-up, but these are found wanting. Whatever the factors contributing to these mixed responses, palliative care services need to consider the most appropriate time to make contact post-death as timeliness is important and also who should make this contact, as consistency appears to be crucial to build rapport and trust in the ability of the service to help. The content of the call/contact should be focused on the specific needs of the bereaved. The assessment of these needs would ideally be initiated by the palliative care service in the pre-bereavement phase of care. Further research is needed to provide an evidence-based for the pre-bereavement assessment of post-bereavement risk, according to the three risk groups outlined in the public health model for bereavement support [18].

In the absence of any formal and systematic assessment of family caregivers’ needs and bereavement risks [11], palliative care services will struggle to make appropriate decisions about providing, or not providing, bereavement support, and if they do for how long [19]. A particular matter that needs to be reviewed is the common practice of assigning bereavement care to a separate team or individual within a palliative care service. There are obvious organisational reasons for doing this, but our findings here underline the importance of having some continuity of relationship, not just continuity of service. That is, the bereavement team may need to consider including someone who was involved in the care of the person who died, or a member of the bereavement team needs to be involved in the care of the family before the death. The advantage a palliative care program has over any stand-alone bereavement program is its role in the pre-bereavement experience of dying people and their social networks. This involvement prior to the death provides opportunities both to assist family and friends in their preparations for death as well as to identify those who may be at risk of greater distress post-bereavement. To enter a bereavement care phase with a new team using generic strategies that invite bereaved people to self-refer is to fail to realise the possibilities inherent in pre-bereavement care.

There was qualitative and quantitative evidence of reliance on support from informal networks such as families, neighbours and friends, also from other services such as the funeral providers and other community based organisations (the Leukaemia Foundation and the Cancer Council), and this was reflected in 68.2% feeling they had received enough support from all community and health providers sources. In fact, we have previously reported that the majority of the bereaved in each of the 3 risk groups accessed support predominantly from family (95%) and friends (88%), followed by funeral directors (79%) and general practitioners (56%) [18], emphasized also in Fig 2. Therefore, palliative care services might do better investing their efforts principally in (a) assessing and supporting family caregivers during the pre-bereavement period and (b) developing community capacity and referral pathways for bereavement care [18, 25, 40, 41].

### Limitations

Although this is not a random sample of the general population, this sample compares well with the UK mortality follow-back survey on cancer [42] in terms of its composition: Responding bereaved females (61% UK, 70% this study), bereaved spouses (38% UK, 41% this study), bereaved sons/daughters (46% UK, 43% this study), the age of the deceased is 77 years for both studies, and proportion male in the deceased (52% UK, 51% this study). Also this sample is drawn from six funeral providers that are based in a mix of metropolitan, regional and rural areas across four Australian states. Although the low response rate in this study is in line with others who relied on postal surveys with no reminder follow up [43], those who did not respond may have had different experiences to those reported in this study. As this is a retrospective study, restricting the time since death to 6–24 months would not have been subject to a large recall bias of events [44]. While some of the wording in the survey questions is not in exact alignment to the wording in the clinical guidelines, it was necessary to adapt them to be lay friendly and we recognise that the responses may have been influenced by the interpretation of the adapted wording. Also the selection of respondents from funeral providers’ database may have influenced the significant number reporting support from these providers.

### Conclusions

Although this national study is based in Australia, it has international implications as our earlier review of palliative care policies and bereavement support practices in several countries (the United States, Canada, the United Kingdom, and Japan) demonstrated similar challenges: questions over providing universal versus targeted support; a lack of clear evidence driving service delivery; informal or no risk assessment; and limited or no evaluation of services [9]. More recent evidence from Europe reinforces our finding that practice of bereavement support in palliative care has only a tenuous relationship with guidelines and assessment tools [10]. While palliative care services offer bereavement support, this is seldom as intentional or targeted as it should be, particularly when the bereavement care phase is separated from the care and support provided prior to the death. Palliative care services should be able to provide more intentional and targeted bereavement care for the population they support. From a public health perspective, however, we have argued elsewhere that, rather than build bereavement services around those who have been in contact with a palliative care program, bereavement services should be developed to serve the whole population of bereaved people. This approach would give priority to the community support needed by most bereaved people, and ensure that professional care supplements, not replaces, the care provided by people’s existing social networks [19]. We believe the findings reported here provide further support for this wider claim. Further research is needed into the attitudes and experiences that underlie the patterns of bereavement support identified in this study.
